# Protocol for the Process Evaluation of the Online Remote Behavioural Intervention for Tics (ORBIT) randomized controlled trial for children and young people

**DOI:** 10.1186/s13063-019-3974-3

**Published:** 2020-01-02

**Authors:** K. Khan, C. Hollis, C. L. Hall, E. B. Davies, D. Mataix-Cols, P. Andrén, T. Murphy, B. J. Brown, E. Murray, C. Glazebrook

**Affiliations:** 1 Division of Psychiatry and Applied Psychology, Institute of Mental Health, School of Medicine, University of Nottingham, Innovation Park, Triumph Road, Nottingham, NG7 2TU UK; 2 NIHR MindTech Medtech Co-operative, Institute of Mental Health, University of Nottingham, Nottingham, UK; 3 NIHR Nottingham Biomedical Research Centre, Nottingham, UK; 4 Department of Clinical Neuroscience, Karolinska Institutet, & Stockholm Health Care Services, Region Stockholm, Stockholm, Sweden; 5 Tic Disorder Clinic, Psychological Medicine Team, Great Ormond Street Hospital for Children NHS Foundation Trust, London, UK; 6 Research Department of Primary Care and Population Health, University College London, London, UK

**Keywords:** Process evaluation, Complex intervention, Mixed methods, Tics, Tourette’s, Children and young people, Randomized controlled trial, Protocol

## Abstract

**Background:**

Process evaluations are an important component in the interpretation and understanding of outcomes in trials. The Online Remote Behavioural Intervention for Tics (ORBIT) study is a randomized controlled trial evaluating the effectiveness of an Internet-delivered behavioural intervention (called BIP TIC) compared to an Internet-delivered education programme aimed at children and young people with tics. A process evaluation will be undertaken alongside the main trial to determine precisely how the behavioural intervention works and ascertain whether, and if so, how, the intervention could be successfully implemented in standard clinical practice. This protocol paper describes the rationale, aims, and methodology of the ORBIT trial process evaluation.

**Methods:**

The process evaluation will have a mixed-methods design following the UK Medical Research Council 2015 guidelines, comprising both quantitative and qualitative data collection. This will include analysing data usage of participants in the intervention arm; purposively sampled, semi-structured interviews of parents and children, therapists and supervisors, and referring clinicians of the ORBIT trial, as well as analysis of qualitative comments put into the online therapy platform by participants at the end of treatment. Qualitative data will be analysed thematically. Quantitative and qualitative data will be integrated in a triangulation approach, to provide an understanding of how the intervention works, and what resources are needed for effective implementation, uptake and use in routine clinical care.

**Discussion:**

This process evaluation will explore the experiences of participants, therapists and supervisors and referring clinicians of a complex online intervention. By contextualising trial efficacy results, this will help understand how and if the intervention worked and what may be required to sustain the implementation of the treatment long term. The findings will also aid in our understanding of factors that can affect the success of complex interventions. This will enable future researchers developing online behavioural interventions for children and young people with mental health and neurological disorders to gain invaluable information from this process evaluation.

**Trial registration:**

International Standard Randomised Controlled Trials Number, ISRCTN70758207. Registered on 20 March 2018.

ClinicalTrials.gov, NCT03483493. Registered on 30 March 2018.

## Background

There is growing interest within health care as to how advances in technology can be used in developing effective treatments for people with psychological and neurological disorders [1]. Although children and young people (CYP) (i.e. individuals up to the age of 18 years) make up a large proportion of the population with psychiatric and neurological conditions [2, 3], there is limited access to evidence-based treatments aimed at reducing symptoms in this population. Access to services for CYP is the lowest amongst all demographics [4] with only 25% of CYP receiving appropriate treatments [5]. Behavioural treatments in particular are desirable and highly recommended by healthcare professionals as a first-line treatment in reducing symptoms in CYP due to the limited side effects relative to pharmacotherapy [6, 7]. However, these treatments are often difficult to access and CYP may avoid face-to-face therapy due to stigmatization [8]. Due to their affinity to technology, a promising development that may benefit CYP are online or digital health interventions (DHI). Randomized controlled trials (RCTs) have shown that DHIs can be effective in treating psychological and neurological symptoms for CYP [9–12] but they can also be ineffective [13, 14]. Hence, before any new DHI is introduced, clinicians, patients and commissioners need robust research to determine efficacy. However, data on efficacy alone is insufficient to inform effective implementation and uptake in routine health care. Data are also required on acceptability, uptake and use of the intervention, including any apparent impact of the digital divide on health inequalities and on the resources and activities required to achieve effective implementation.

Little is known about how, and for whom in particular, DHIs work and what makes them effective in one context and not another and the barriers to effective implementation [15, 16]. The UK Medical Research Council (MRC) has developed specific guidelines for conducting a process evaluation of complex interventions to assess quality of implementation (fidelity), dose, reach and adaptations and to analyse causal mechanisms and identify any contextual factors [17]. Process evaluations can therefore aid interpretation and understanding of trial outcomes and inform future refinements of the intervention under study.

Grant et al. [18] have identified the importance of outlining process evaluation methodology a priori and consider the publication of process evaluation protocols as “best practice” in order to improve trial quality. Despite the increasing popularity in conducting process evaluations of complex interventions [16, 19] and the aforementioned importance of publishing protocols*,* explicit guidelines for publishing process evaluation protocols are limited [20].

Using previously published process evaluations of complex interventions protocols as a guide [21, 22], here we outline the methodology and describe the planned process evaluation of the Online Remote Behavioural Intervention for Tics (ORBIT) trial.

### The ORBIT intervention

The ORBIT trial and its BIP TIC intervention have been described in detail previously as part of the main trial protocol [23] (03/01/2019; version 3.0), a brief summary is given to provide context to the process evaluation design. The ORBIT trial is a 10-week, parallel group, single-blind RCT with an internal pilot. ORBIT aims to evaluate the efficacy of an online, remote, therapist-supported and parent-guided behavioural intervention for tics, which was initially developed and piloted in Sweden and called BIP TIC [24]. The comparator is an online, remote, therapist-supported and parent-guided psychoeducation programme for tics. Participants will be recruited from clinics, Patient Identification Centres (PICs) across National Health Service (NHS) Trusts, or from the two study sites involved in the trial (Queen’s Medical Centre (QMC), Nottingham and Great Ormond Street Hospital (GOSH), London), or via a tic disorder charity (Tourettes Action), the ORBIT study website or social media. Participants need to be 9–17 years old and be suspected or confirmed as having Tourette syndrome (TS) or chronic tic disorder (CTD) and must not have had any form of behavioural treatment for tics in the last 12 months or a change in medication for tics in the previous 2 months. Participants will be followed up mid treatment and at 3, 6, 12 and 18 months post-randomization.

Participants will be randomized to one of two groups. The intervention group will receive 10 self-help modules of behavioural therapy delivered over a period of 10–12 weeks, which will be accessed via a secure online platform [24]. The behavioural therapy will follow evidence-based exposure and response prevention (ERP) therapeutic principles, whereby patients learn strategies for managing their tics through allowing premonitory urge sensations to come to the fore and actively tolerate the premonitory urges and suppress their tics. In doing so, the child masters their ability to tolerate the urge, control their tics, and is able to do so for an increasing amount of time in a hierarchical manner. The child also receives education about tics for the family and others, such as teachers, friends and family. Throughout the 10–12 weeks, participants will have access to a therapist and their role is to encourage participants to engage with the treatment content and its homework assignments, and answer any queries that participants may have. The parent components contain information about how to support their child and various coping strategies for themselves. Previous studies have shown that ERP is effective in reducing tics [25, 26], with European clinical guidelines [25] and a National Institute of Health Research Health Technology Assessment Evidence Synthesis [27] recommending that behavioural therapy should be offered as a first-line intervention for tics in CYP. The primary outcome measure is the severity of tics as measured on the Total Tic Severity Score (TTSS) subscale of the Yale Global Tic Severity Scale (YGTSS) [28]. The target sample size for the ORBIT trial is 220 (110 in the intervention arm and 110 in the control arm).

Overall, the ORBIT trial aims to evaluate the clinical effectiveness of an online behavioural treatment for CYP with tics compared to online tic-related education in reducing tics, as measured by the YGTSS TTSS. Furthermore, the trial aims to evaluate the cost effectiveness of the online treatment and to estimate the longer-term impact on patient outcomes and health service costs.

### The ORBIT process evaluation aims and objectives

The aim of the ORBIT process evaluation is to understand the causes of the observed behaviour change using data obtained from the RCT, and in particular, to explore the fidelity of intervention delivery, acceptability of the intervention and reasons for observed variation in uptake and use, and to consider the resources and implementation processes required.

Specific objectives are:

1. To assess the fidelity, reach and dose of intervention delivery.
2. To explore whether any of the intervention features were adapted for individual needs enabling potential recommendations for adaptations.
3. To explore BIP TIC from the perspective of children, parents, therapists and clinicians in order to gain a deeper understanding of potential mechanisms underlying participant’s behaviour change whilst probing for any unexpected consequences.
4. To evaluate any factors external to BIP TIC that may have affected delivery (i.e. the environment and its characteristics) or whether its mechanisms of impact worked as intended.
5. To consider the resources and implementation processes required for effective implementation, uptake and use of the intervention.

The design of this process evaluation is guided by MRC directives on the process evaluation of complex interventions [17]. The MRC outlines three essential components in understanding how outcomes are achieved: implementation, mechanisms of impact and context. The application of these guidelines in the context of the ORBIT trial will be as follows:

1. Implementation: an exploration as to how delivery of BIP TIC was achieved by examining quality (fidelity) and quantity (dose) of what was implemented. The structures and processes through which BIP TIC was delivered as intended, any adaptations made, and establishing the extent to which BIP TIC reached its intended audience (reach).
2. Mechanisms of impact: an examination of the causal mechanisms through which BIP TIC produces change by understanding how participants interact with the intervention. This also allows for an identification of any unexpected pathways and consequences.
3. Context: an exploration of any factors external to BIP TIC, which may have influenced its implementation (e.g. comorbidities, home life for the family, school life for the child, system factors in health services). MRC guidelines outline that a process evaluation should address how context affects implementation and outcomes (i.e. change). They further suggest that when investigating impacts of context on outcomes, it is helpful to relate contextual variations to a priori hypothesised causal mechanisms, or those emerging from qualitative analysis, in order to generate insights into context-mechanism-outcome patterns. Thus, in order to explore context, we will be as flexible as possible in data analysis.

MRC guidance on the development and evaluation of complex interventions notes that identifying and developing a theoretical understanding of the likely process of change is a key early task for developing a complex intervention or evaluating one that has already been developed. MRC guidelines stipulate an important component of a process evaluation is to outline the processes of the intervention and the outcomes it aims to achieve by means of a logic model. The logic model for the study is shown in Table 1.

**Table 1:** Logic model for the BIP TIC intervention

Problem	Delivery mechanisms	Intervention (What is to be implemented) How delivery achieved	Mechanisms of impact	Intended outcomes	Impact
Growing demand for behavioural therapy as a first line treatment Lack of specialised care for CYP with tics	ERP Education on tics and comorbidities	Therapist reinforcement Follow-up sessions Therapist support Knowledge about tics and management	How people feel about BIP TIC Motivation levels Treatment credibility	Reduced tics Reduced co-morbid psychological symptomology of psychiatric condition Increased parental and CYP awareness and knowledge Improved function (e.g. school, social relations, leisure activities)	Improved provision of care for CYP with tics Increase in behavioural therapy as a first line treatment Health economic aspects
	Parent resources	10 modules at weekly intervals	Unanticipated consequences		
	Therapist contact	Rewards Regular practice	Mediators		
		Parental support			

*CYP children and young people, *ERP* exposure and response prevention

### Overall design

The overall design of the ORBIT process evaluation is a mixed-methods study using purposively sampled qualitative data together with quantitative data from the trial. This will involve semi-structured interviews with children, parents, therapists and supervisors and clinicians, and analysis of online feedback from participants together with data from the online platform, such as total therapist time, number of chapters viewed and number of log-ins.

**Fig. 1:**
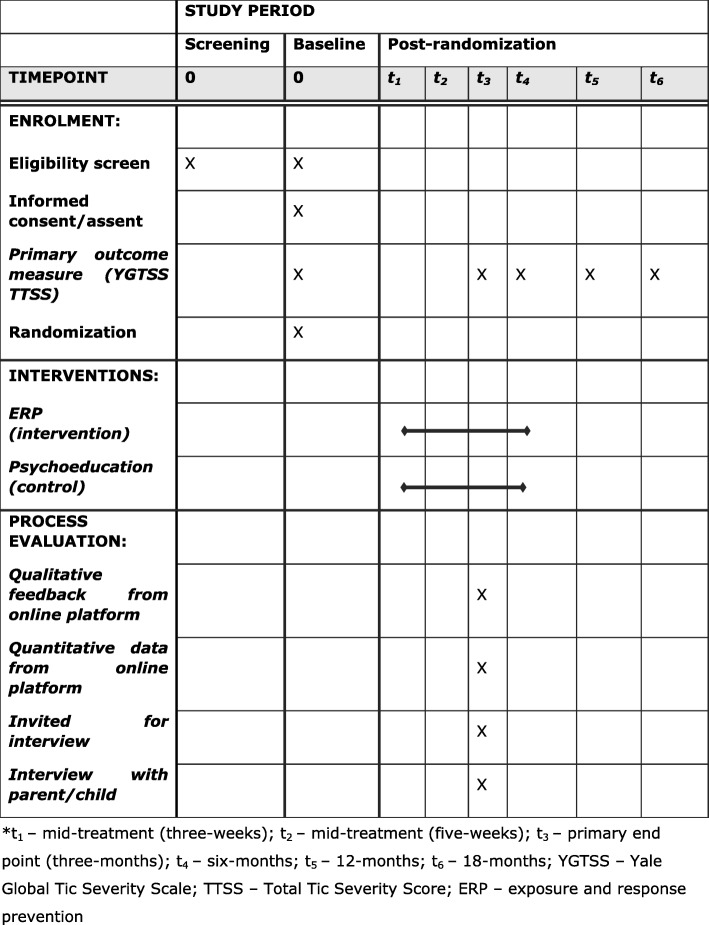
Schedule of ORBIT and process evaluation procedures. *t_1_, mid-treatment (3 weeks); t_2_, mid-treatment (5 weeks); t_3_, primary end point (3 months); t_4_, 6 months; t_5_, 12 months; t_6_, 18 months; YGTSS, Yale Global Tic Severity Scale; TTSS, Total Tic Severity Score; ERP, exposure and response prevention

The schedule of the ORBIT process evaluation procedures is displayed in Fig. 1. In Additional file 1 a populated Standard Protocol Items: Recommendations for Interventional Trials (SPIRIT) checklist is provided [29] and in Additional file 2 a CONSORT-EHEALTH: Improving and Standardizing Evaluation Reports of Web-based and Mobile Health Interventions is provided [30].

Ethical approval for the process evaluation was obtained from North West - Greater Manchester Central Research Ethics Committee as part of the ORBIT trial (REC: 18/NW/0079).

### Qualitative data collection

Qualitative data will be collected by interviewing participants in the BIP TIC intervention (both CYP and parents, either separately or as a dyad), therapists and supervisors and referring clinicians. Interviews with therapists and supervisors involved in the ORBIT trial will be conducted early in the trial and near the end of recruitment in order to gain an understanding of their experience at different time points. All interviews will be conducted either by telephone or by the WebEx videoconferencing application. In addition, at the end of treatment participants are asked within the BIP TIC platform questions including what the most important thing they have learnt from treatment, how the treatment has helped, if the treatment caused any difficulties to participants, and any other comments they may wish to add. These data will be put into BIP TIC and then will be exported to an excel spreadsheet and content analysis will be performed.

### Sampling and recruitment for interviews

#### Children and parents

In line with previous literature [31, 32], four semi-structured interview schedules were developed (see Additional file 3). The child and parent interview schedules were drafted and underwent revision from the main researcher and three academics. Questions include (1) how they found out about the ORBIT trial; (2) why they took part; (3) their initial expectations; (4) their views of the content, structure, and the different chapters of the online programme; (5) what impact the therapy had, if any, on their tics; (6) what they found most and least helpful; (7) barriers to participation; (8) how they felt about communicating with their therapist; (9) if they would alter anything about the programme and (10) their recommendations for improvement of the interventions and their overall experience of participating in the trial. The revised drafts were sent to two dyads of the Patient and Public Involvement (PPI) group - including two children with tics - for feedback and were revised accordingly.

All interviews will be carried out with CYP and parents of CYP following completion of the intervention at the 3-month (primary end-point) follow-up assessment in the main trial. Recruitment for the interviews began in August 2018 through the following methods:

- Following completion of the primary end-point, the researcher conducting the follow-up assessment asks participants if they are willing to be contacted about taking part in an interview. If the participant agrees, the researcher informs the process evaluation researcher who makes contact with the family.
- Researchers at both QMC and GOSH arrange a convenient date, time and method for interviewing the participants who agree to this following their primary end-point follow-up assessment.
- A proportion of the participants are to be contacted by telephone following their primary end-point assessment by the main researcher of the process evaluation.

Participants will only be contacted if they gave explicit written consent to participate in an interview for the ORBIT trial and, for a child under 16 years old, assent was obtained with parental consent (see Additional file 4). Participants will be purposively sampled with the intention of collecting data from a diverse cohort to obtain varying views on the intervention. This will include ensuring perspectives from a range of ages, gender and ethnicity, and levels of interaction with the intervention are voiced. We anticipate that this sampling strategy will result in sufficient heterogeneity to provide examples of both relatively poor and relatively good adoption, delivery and maintenance, and will allow us to identify barriers and facilitators to implementation and to generate hypotheses about factors that may be associated with differing outcomes for CYP in the intervention arm.

The target sample size for participant interviews is > 20 CYP and > 20 parents of CYP. This will ensure that data reach a level of saturation [33] and enable diversity of views.

#### Therapists

The therapist interview schedules were drafted and were revised by the main researcher and three academics, with input from a therapist and clinical researcher with specific expertise in the field. Therapist questions include (1) their role on the ORBIT trial; (2) how they found out about ORBIT and why they became involved; (3) what specific skills they felt a therapist needed for the programme; (4) any training needs identified; (5) how they managed ORBIT around other commitments; (6) their experiences of receiving/giving supervision sessions; (7) if the therapy is being delivered as planned; (8) their experiences of interacting with participants; (9) their views on the two trial arms and (10) and their recommendations for future use.

Therapists will initially be interviewed individually early in the trial (halfway through the study) and then interviewed again near the end of the trial. This will allow for a range of experiences at different time points to investigate trial progression. The target number of therapist interviews is > 5, of which 2 will be interviews with supervisors.

#### Clinicians

Clinicians refer to any healthcare professional (usually a physician) who were responsible for referring participants to the ORBIT trial. Whilst they were not explicitly involved in the ORBIT trial, the main purpose of interviewing them was to gain their views on potential implementation in routine care. The clinician interview schedules were drafted and underwent revision from the same team and were guided by normalization process theory (NPT) [34, 35]. As the purpose of the clinician interviews is to explore their views about the feasibility of integrating the intervention into everyday practice, including any potential barriers to or facilitators of this, the NPT framework approach seemed the most appropriate. The clinician interview schedule questions aim at eliciting information on how they became involved in the ORBIT trial and why, their experience of recruiting for the trial including factors that affected recruitment, and how the NHS could incorporate the intervention into everyday practice. Clinicians will be purposively selected from the PIC sites involved in recruiting for ORBIT and the target number of clinician interviews is > 5.

### Quantitative data collection

Online data will be collected and recorded from participants throughout the trial. These include the following measures: total therapist time; therapist time specific to each therapist; therapist time specific to each child and parent; total number of characters submitted by child and parent (as part of communication messages via the online system); total number of logins for child and parent; average time between each login (in days) for child and parent; average pages visited per login for child and parent and the five most frequently visited pages per child and parent. These data will be amalgamated and entered into a centralised online database whereby the main researcher will then extract this data for analysis as part of the process evaluation.

#### Trial data

As part of the quantitative measures for the process evaluation, we will also extract and analyse change in YGTSS TTSS from baseline to the primary end-point, which will be used to inform behaviour change. As mentioned, this is a key component of the MRC guidance on process evaluations. Demographic data, overall symptom improvement as measured on the Clinical Global Impressions Scale (CGI) for improvement [36], depressive symptoms at baseline as measured on the Mood and Feelings Questionnaire (MFQ; child-completed version) [37] and service-use data as measured by the modified Child and Adolescent Service Use Schedule (CA-SUS) [38] will also be analysed. These data will be used to measure context and the mechanisms of change. The target sample size for all quantitative data is 110 participants. Table 2 presents a summary of the explanatory data sources that will be used to inform each component of the process evaluation.

**Table 2:** Process evaluation components, areas of research, explanatory data and outcomes

Process evaluation components	Research questions	Explanatory data	Outcomes
Implementation (what is implemented and how?)	➢ Fidelity of implementation ➢ Dose of intervention delivered ➢ Adaptations ➢ Reach	➢ Therapist contact/time (*n* = 110) ➢ BIP TIC adherence (*n* = 110) ➢ Usage metrics (*n* = 110) ➢ Clinician (*n* > 5), children and parent (*n*= > 20), therapist (*n*= > 5) interviews	➢ Engagement and satisfaction with intervention
Mechanisms of impact (how does it produce change?)	➢ Mediators and moderators ➢ Unexpected pathways and consequences	➢ Usage metrics ➢ Therapist contacts ➢ Clinician, children and parent, therapist interviews	➢ YGTSS TTTS change
Context (how do factors external to the intervention affect implementation and change?)	➢ Factors related to improvement in YGTSS TTSS, fidelity of delivery	➢ Demographic data ➢ Clinician, children and parent, therapist interviews ➢ Service use ➢ Comorbidities ➢ Baseline severity of tics	➢ YGTSS TTTS change ➢ Engagement with intervention

^a^*YGTSS* Yale Global Tic Severity Scale, *TTSS* Total Tic Severity Score, *BIP TIC* Internet-delivered behavioural intervention

### Data analysis

Qualitative data will be exported and analysed in QSR International’s NVivo 12 Software [39] and quantitative data will be exported and analysed in SPSS (version 25.0) [40]. Process evaluation data will be analysed autonomously of the main outcome data of the ORBIT trial.

### Qualitative data analysis

All interviews will be recorded either by the WebEx videoconferencing application or by Dictaphone and then transcribed verbatim. Transcripts will be checked for accuracy against the recordings, with any corrections made as appropriate. Prior to importing transcripts into QSR NVivo 12, any reference to places, clinicians, therapists, and/or family members that may reveal participants’ identity will be redacted, and all participants’ names will be anonymised. The interviewer will take notes during all interviews.

As the process evaluation is a combination of exploration and description, thematic analysis will be used to identify, analyse and report patterns within the transcribed interviews. Thematic analysis is widely used within the field of psychology and is considered the most flexible qualitative analytical process [41]. More broadly, the framework method [42] of analysis will be employed, as it is most commonly used for the thematic analysis of semi-structured interviews [43]. Moreover, Ritchie and Spencer [42] outline four types of research questions that they believe framework analysis can helpfully address: (1) contextual - identifying the form and nature of what exists (e.g. what is the nature of people’s experience?); (2) diagnostic - examining the reasons for, or causes of, what exists (e.g. why are services or programmes not being used?); (3) evaluative - appraising the effectiveness of what exists (e.g. what affects the successful delivery of programmes or services?) and (4) strategic - identifying new theories, policies, plans or actions (e.g. how can systems be improved?). As the process evaluation covers all of these questions, we feel this is the appropriate methodology to use.

Ritchie and Spencer [42] suggest five key stages of framework analysis: familiarisation, identifying a thematic framework, indexing, charting and mapping and interpretation. During the familiarisation stage, the main researcher will immerse himself in the data by listening and/or watching back the interviews, reading transcriptions and studying observational notes whilst listing key ideas and recurring themes. The data will then be analysed to identify key issues, concepts, themes, and sub-themes drawing on both a priori and emergent issues. Next, the transcripts will be coded and indexed into framework categories by systematically applying the thematic framework to each interview. The indexed data will be summarized for each category and organised in chart form. This process will involve working through each framework category, summarizing all data that have been indexed to that category, and then providing a summary for each category for each participant, using headings and subheadings. Consequently, key characteristics of the holistic dataset will be mapped and interpreted. An independent coder will double-code a subset of transcripts to identify emergent patterns and themes relating to participants’, therapists’ and clinicians’ experiences of the ORBIT trial. Charted data will be annotated independently and the findings discussed, which will allow for refinement and amendment of data in an iterative process. Once confidence in the congruity and meaningfulness of interpretation is established between researchers, we will review the remaining interviews to establish whether our understanding has reached acceptability.

The large amount of data collected for the process evaluation encouraged us to use computer-assisted qualitative data analysis software (CAQDAS). The CAQDAS package, QSR NVivo 12, is fully integrated with framework analysis and this will be used to categorise data and document any themes and sub-themes. Online feedback given by participants at the end of therapy will be analysed using content analysis and integrated into the aforementioned framework.

### Quantitative data analysis

Quantitative data from the online platform will be analysed descriptively by calculating total numbers and percentages and mean and standard deviation or if the data are not normally distributed, the median and range. This will provide information on intervention delivery, including the implementation of different components and fidelity. The independent samples *t*-test and the chi-squared test will be used to explore any significant differences within the intervention group. Data that are not normally distributed, will be analysed using non-parametric alternatives (i.e. Kruskal–Wallis H and Mann–Whitney U tests), using a significance level of *P* < 0.05.

### Mixed methods analysis

Qualitative and quantitative data will be analysed separately and then mixed during analysis in a methodological approach known as triangulation [44]. Both qualitative and quantitative data will be given equal importance, as both sets of data are central to addressing the research questions posited by the process evaluation. A Good Reporting of a Mixed Methods Study (GRAMMS) [45] checklist is provided in Additional file 5.

Qualitative data and preliminary qualitative analysis will be coded synchronously with the analysis of descriptive statistics of participants’ online data. Thus, the descriptive data will aid in the refinement and amendment of questions central to qualitative data collection. In other words, key themes may emerge from the quantitative data, which could then be further explored or clarified from qualitative data, and vice versa. The main researcher will integrate and compare outcomes from the various datasets guided by the triangulation protocol. The aim of this is to create a matrix of converging datasets to assess outcomes where there is agreement or dissonance, and where themes or outcomes emerge in one dataset but not another. Once the matrix of outcome synthesis from the various datasets is finalised, it will be used to emphasise the mechanisms of impact, implementation fidelity and, more broadly, explain the outcomes of the trial.

### Integration of findings

The process evaluation data will be analysed prior to knowing the main ORBIT trial results, with the two analyses being independent of each other. The ORBIT trial team will be unaware of the findings of the process evaluation until the primary outcomes from the main trial have been analysed. Once both trial and process evaluation analyses are complete, combined qualitative and quantitative data may aid in the development of hypotheses about the potential successful implementation in one context over another and how and why some components were delivered successfully and others were not. Furthermore, the analysis of different components may aid in the identification of causal mechanisms and how and why individual intervention components were more effective than others. Following quantitative analysis of ORBIT trial data, qualitative data from the process evaluation can potentially be used to help explain the outcomes of the trial. Additional analyses can then be conducted to test hypotheses emanating from integration of process evaluation data with trial outcomes, drawing together the findings to understand why the intervention worked (or not), context and implications for further dissemination to improve provision of care for CYP with tics.

## Discussion

This protocol outlines the rationale, design and methodology for the planned mixed methods process evaluation of BIP TIC, a complex online intervention for CYP with tics. The process evaluation is designed to explore the implementation of the online intervention and provide a holistic view of trial outcomes. By explicitly outlining our process evaluation methodology, guided by MRC framework of complex intervention trials [17], this paper adds to the literature on process evaluation protocols using a mixed methods design. In doing so, this will improve the integrity of this process evaluation and, as mentioned, there is growing emphasis on the importance of publishing process evaluation protocols in advance to improve overall trial quality and reporting [18].

The combined qualitative and quantitative process evaluation data will support the homogenous interpretation of the main outcome data from the ORBIT trial. By illuminating how and why BIP TIC was effective or not, the process evaluation will help elucidate a holistic view of the intervention. Moreover, understanding the mechanisms of impact and any contextual factors, these data will augment the dissemination plan and may support the long-term implementation of the intervention. The process evaluation will also offer insight into digital interventions and may inform future development of such health technologies.

### Strengths and limitations

Conducting the process evaluation will contribute to explaining the overall findings of the main RCT: the factors underlying positive and negative effects of different aspects of BIP TIC. For example, if there were certain negative outcomes from using BIP TIC, the process evaluation will be an invaluable resource in elucidating whether the intervention was inherently inadequate, if there was a failure of implementation and if this was related to participants (e.g. lack of motivation) or contextual factors (e.g. pre-existing beliefs about online therapy). This would help to improve the intervention progressively.

In contrast, if there were positive outcomes from using BIP TIC, the process evaluation will identify the core components that made the intervention a success. For example, if it was determined that an essential component for promoting participants’ adherence to the intervention was the use of parental support and therapist encouragement, these findings will be crucial to the development and implementation of future digital programmes aimed at CYP with tics.

By collecting data from a range of relevant stakeholders (e.g. parents, children, therapists and supervisors and clinicians) and combining quantitative and qualitative data, we will gain a holistic understanding of the mechanisms underlying the impact of the intervention. Furthermore, the proposed sample size is adequate to capture a comprehensive overview of perspectives, generating rich data and analytical depth.

One potential limitation arising from this is that the majority of participants who drop out of treatment are more likely to refuse to be interviewed, which could lead to a more positive overall evaluation of the intervention. We will attempt to overcome this by making a more concerted effort to recruit participants who drop out of treatment or, if this is not possible, those who complete fewer modules. The main limitation in terms of future implementation is that the environment/context will be heavily influenced by this study being an RCT. It would arguably be more appropriate to conduct a parallel implementation study; however, lack of resources prohibit this.

### Trial status

The trial and recruitment of participants for the process evaluation is ongoing.

## Supplementary information

**Additional file 1.** SPIRIT 2013 Checklist: Recommended items to address in a clinical trial protocol and related documents.

**Additional file 2.** CONSORT-EHEALTH (V 1.6.1) Submission/Publication Form.

**Additional file 3.** Child, parent, therapist and clinician interview schedules.

**Additional file 4.** ORBIT Consent and Assent forms.

**Additional file 5.** Good Reporting of a Mixed Methods Study (GRAMMS) checklist.

## Data Availability

Not applicable.

## Abbreviations

BIP TIC: Internet-delivered behavioural intervention
CTD: Chronic tic disorder
CYP: Children and young people
DHI: Digital health intervention
ERP: Exposure and response prevention
MRC: Medical Research Council
NHS: National Health Service
ORBIT: Online Remote Behavioural Intervention for Tics
PIC: Patient Identification Centres
PPI: Patient and public involvement
RCT: Randomized controlled trial
SPIRIT: Standard Protocol Items: Recommendations for Interventional Trials
TS: Tourette syndrome
TTSS: Total Tic Severity Score
YGTSS: Yale Global Tic Severity Scale
