# Supplementary material for: Protocol for the Process Evaluation of the Online Remote Behavioural Intervention for Tics (ORBIT) randomized controlled trial for children and young people
Source: Trials. 2020 Jan 2;21:6. doi: 10.1186/s13063-019-3974-3 (PMC6941346; doi:10.1186/s13063-019-3974-3)
Supplement: Supplementary file 3 — Additional file 3. Child, parent, therapist and clinician interview schedules. [file 13063_2019_3974_MOESM3_ESM.docx]

**CHILD INTERVIEW SCHEDULE**

**Preamble**

*If interview is conducted face to face then child will have the option of having their parent sit with them.*

*If interview is conducted over the phone the child can have the option of placing the call on loudspeaker with their parent listening in.*

*In many places two options of words are given, as one word will be chosen over another depending on the child’s age and linguistic ability.*

- Check that the interviewee has received the information sheet, they/their parent (*depending on age*) has initialled the box stating they are happy to be contacted for an interview on the consent/assent form, understands the ORBIT project and his/her role in it
- Make sure refreshments available in room and that room is set-up ready for interview.
- Explain that:
  - The aim of the ORBIT study was to see whether online therapy can help children and young people with tics.
- The research team is speaking to many people involved in ORBIT e.g. parents, therapists, and clinicians
- We are interested in your experiences and thoughts about ORBIT, so please give honest answers, as both positive and negative feedback will help us improve the therapy. You will be asked questions about the ORBIT therapy.
- However, we will put all the data that we collect together in a report to give us an overall picture of ORBIT and no one will be named in the report or know what you answered to the questions, for example, “A young person/child commented that…”

*Ask*: Do you have any initial questions about the project?

**Ethics**

- Remind interviewee:
- The interview will take about 30 minutes
- You do not have to answer any questions that you are not comfortable with and there are no ‘right’ or ‘wrong’ answers
- You can stop at any time, no explanation needed
- If you need a comfort/loo break, please just say, that’s absolutely fine
- If any question doesn’t make sense, ask me to explain

With your permission we are going to record the interview (audio only, on a Dictaphone) so that we can focus on what you are saying. This will be written out by a member of the research team or a company we know well. If you feel more comfortable that we write out the interview rather than the company then we will be happy to do so.

We will delete any mention of places, clinicians/therapists/family members that may give away yours (or others) name during writing.

The original writing will be put on a password protected hard drive and no one other than members of the research team will be able to view this.

The things you say in the interviews may be used in written reports, published articles and presentations including online but we will never use your name or any other information that may give away who you are.

*Ask*: Do you have any questions about how we use your comments? Please feel free to ask anything however small it may seem at this stage or at any time later.

*Ask*: Is it okay to record the interview?

- If participant not satisfied: answer any questions they have. If they do not want to participate, thank them for their time and finish the interview at this point.

**Explain procedure**

I will begin the interview with my name, the date, time and the code we have for you - this is just to keep the recordings organised. All your details will be hidden when the interview is written out. The first part will be a little about yourself and your tics, followed by general questions about the ORBIT project such as what you thought about the study and questionnaires, then moving on to the ORBIT therapy and then ending with any recommendations and your overall experience of being involved in ORBIT.

*Ask*: Do you have any questions before we start?

*Ask*: Is it okay for me to start recording now?

*State researcher’s name, date, time, and identifying code (for data management)*

**Warm up**

1. At the beginning of the study, we sent out information about this study to your mother/father/parents (*personalise according to who the information was sent to*), do you remember if they spoke to you about it and did they speak to you about whether you wanted to participate or not?
2. Please tell me a little about yourself

- Hobbies/interests?
- Family, things you like to do together?
- School life

1. Can you tell me what tics you have?
   **Prompt**

- Vocal/motor?
- Simple/complex? (*Give examples if child does not know the difference)*
- How often do they happen?
- How do they make you feel?
- Impact?

1. Have you noticed any difference in your tics in the last 3 months?

**Prompt**

- Type/frequency/severity

First I am going to ask you questions about being part of the ORBIT study, including how you felt about this and how you found the questionnaires:

**Questions about ORBIT as a research project**

1. What did you think about the sound of the ORBIT study?

- Who told you about it? (TA? Clinician? Parent?)
- How did you feel about taking part?

1. What did you think of the stuff you had to do before the therapy began?

- Face to face meeting (baseline appointment)
- Information sheet
- Consent/assent form
- Did you get any help from your parent?

1. Was it clearly explained to you that you would be put in to one of two groups?

- How did you feel about this?
- How did you feel about the group were put in?

1. Thinking about what was expected of you during the ORBIT study:

- Can you remember filling in questionnaires? What did you think of them?
- Was the study clearly explained to you?
- How did you feel about online questionnaires?
- How did you feel about face to face questionnaires?
- Which did you prefer?
- Has it been okay to manage or a lot of effort? How much help did you need?

Thank you for these answers, that’s been really helpful. I’d now like to move on and ask you some questions about how you found the ORBIT therapy:

**Questions on ORBIT therapy**

1. I am now going to go through the different parts of the ORBIT therapy and I would like you to tell me how you found them:

- How easy was it to log on?
- Did you find it difficult to use anything in the online therapy?
- What did you think of the lay out/graphics?
- What did you think of the content/things included?
- Anything that worked particularly well? Anything that could have been improved?
- Did it make sense to you as you did it?
- Any help needed from parents?
- How did you link ORBIT therapy into everyday life?

1. How did you use the ORBIT therapy?

- Did you do the chapters on your own?

1. Was there anything that stopped you from doing the ORBIT therapy?
2. Overall, what did you think about the different sections of ORBIT?

- Any sections that you particularly liked or engaged with?

**Prompt**: why?

- Any sections that you did not engage with/found hard?
- Do you think the therapy was too long/too short/just right?
- What about the rewards – did they help/motivate you?

You are now half way through the questions so I want you now to think about the ORBIT therapy and I want to ask you some questions on the impact/effect it has had on you:

1. Before you started the ORBIT therapy, how did you think it would help your tics and everyday life?

- How good did you expect it to be in reducing/cutting down tics?
- Did you expect benefits/to help in any other areas of life?

1. How much do you think the ORBIT therapy has helped you with your tics and in everyday life?
2. Which parts of the therapy were particularly helpful to you?
3. Did you have any difficulties with the ORBIT therapy?

- If so, what were they?
- Technological difficulties? Did you manage to sort them out?
- What did you use to access ORBIT? i.e. tablet/PC/smartphone etc.

1. Did you follow the ORBIT therapy exactly as it was laid out/did you do the online chapters in order?

- Did you adapt/change anything as you went along?

1. Would you change anything about the ORBIT therapy?

- Should we add any information?
- Anything that was not needed?

1. How did you feel about talking with (*name of therapist*)?

- How often did you contact them?
- Was it helpful?
- How did you contact them? Through email? Through ORBIT website? Phone?
- Which way of contacting a therapist do you prefer?

1. How did you feel about getting the therapy online instead of face to face?

**Prompt**: how would you have preferred?

Thank you for answering those questions. We only have a few questions left now and these will focus on the future of the ORBIT therapy:

**Future Direction**

1. If the ORBIT therapy is found to be effective/helpful are there any changes we could make before it is offered to other children and young people?
2. Are there other ways that could be used for giving ORBIT therapy to people?

- Skype? WhatsApp?
- Any other apps or forms of technology?

1. Overall, how did you feel about the experience of taking part in this study?
2. Would you recommend ORBIT therapy to other children and young people with tics?

**Prompt** for clarification of response.

**End of questions**

That reaches the end of the interview and questions I wanted to ask you.

Thank you so much for your time.

- Do you have anything else you wish to speak about that hasn’t been mentioned?
  - Let interviewee talk if they have anything else to add
  - If nothing else – then close interview

If you are okay to end the interview there, I will switch the Dictaphone off.

*Switch Dictaphone off*

**Debriefing**

- Ask how they are feeling – whether anything in the interview has troubled them or distressed them or if anything requires clarification
- They or their parents can email me if they have any follow up questions
- Thank them again, and ask if they are feeling okay to end interview here.

**PARENT INTERVIEW SCHEDULE**

**Preamble**

- Check that the interviewee has received the information sheet, has initialled the box stating they are happy to be contacted for an interview on consent form, understands the ORBIT project and his/her role in it
- Make sure refreshments available in room and that room is set-up ready for interview.
- Explain that:
  - The aim of the ORBIT study was to investigate whether treatment delivered online can help children and young people with tics.
- The research team is speaking to a range of people involved in ORBIT e.g. children who participated, therapists, and clinicians
- We are interested in individual experiences and thoughts about ORBIT, so please give honest responses, as both positive and negative feedback will help us improve the intervention. Explain that they will be asked questions relating to their expectations of ORBIT, their thoughts on the treatment, impact on child, level of engagement, difficulties and challenges experienced, and any recommendations they may have
- However, we combine all the data we collect to provide an overall picture of ORBIT and its implementation and any comments in the report are attributed very generally, for example, “A parent commented that…” All comments/opinions will be strictly confidential.

*Ask*: Do you have any initial questions about the project?

**Ethics**

- Remind interviewee:
- The interview will take about 30 minutes
- You do not have to answer any questions that you are not comfortable with and there are no ‘right’ or ‘wrong’ answers
- You can stop at any time, no explanation needed
- If you need a comfort/loo break, please just say, that’s absolutely fine
- If any question doesn’t make sense, ask for an explanation

With your permission we are going to record the interview (audio only, on a Dictaphone) so that we can focus on what you are saying. The interviews will be transcribed by a member of our research team or an approved company. If you do not wish for interview to be transcribed by the approved company then please let us know and we will transcribe internally instead.

We remove any reference to any places, clinicians/therapists/family members that may give away your (or others) identity during transcription.

The original transcription will be stored on an encrypted hard drive and no one other than immediate members of the research team can access this.

Anonymised quotes from transcripts will be used in written reports, published journal articles and presentations including online. Again, any reference to places/family members/clinicians and so on will be removed.

*Ask*: Do you have any questions about how we use your comments? Please feel free to ask anything however minor it may seem at this stage or at any time later.

*Ask*: Is it okay to record the interview?

- If participant not satisfied: answer any questions they have. If they do not want to participate, thank them for their time and finish the interview at this point.

**Explain procedure**

I will begin the interview with my name, the date, time and the identifying code we have assigned to you and your child - this is just to keep the recordings organised. All your details will be anonymised when the data is transcribed. The first part will be a little about yourself and any other studies on tics you may have been involved in, followed by general questions about the ORBIT project such as how you were recruited and expectations, then moving on to the ORBIT treatment more specifically and then ending with any recommendations and your overall experience of being involved in ORBIT.

*Ask*: Do you have any questions before we start?

*Ask*: Is it okay for me to start recording now?

*State researcher’s name, date, time, and identifying code (for data management)*

**Warm up**

1. Please tell me a little about yourself and your family

**Prompt** (if no response)

- Things you like to do together?

1. Have you and your child ever taken part in any other studies on tics?

- What did that involve?

1. Can you tell me what tics your child has?

Prompt

- Vocal/motor?
- Simple/complex (*give examples if unsure of the difference*)
- How often do they happen?
- How do they make you feel?

1. Have you noticed any difference in your child’s tics in the last 3 months?

**Prompt**

- Type/frequency/severity

First I am going to ask you questions about being part of this research trial, including how you felt about this and how you found the questionnaire completion:

**Questions about ORBIT as a research project**

1. How did you find out about the ORBIT project?

- Who told you about it? (TA? Clinician? Friend?)
- What did you hope to get out of the trial from both you and your child’s point of view?
- What were your initial thoughts about the ORBIT project?

1. Why did you get involved in this project?
2. What did you think of the way you were approached to take part?

- What did you think of the initial telephone screening?
- What did you think of the face to face meeting (baseline appointment)?
- Have you any comments on the information sheet and consent?
- Anything that you would have liked to be done differently?

1. Was it clearly explained to you that you and your child would be allocated to one of two groups? One to learn strategies on how to control tics and one to receive information on tics?

- How did you feel about being “randomised”?
- How did you feel about the group that you/your child was allocated?

1. Thinking about what was expected of you during the ORBIT trial:

- Can you remember what the study involved for you in terms of completing questionnaires?
- Was the ORBIT trial clearly explained to you?
- How did you feel about online questionnaires?
- How did you feel about face to face questionnaires?
- Which did you prefer?
- Did you expect it to take a lot of effort to get your child to engage?

Thank you for these answers, that’s been really helpful. I’d now like to move on and ask you some questions about how you found the ORBIT therapy:

**Questions on ORBIT therapy**

1. How did you feel about the delivery of the parents’ materials in ORBIT?

- How did you feel about the logging on process?
- Was it technically easy to use/easy to understand?
- How did you feel about how the material was presented?
- What did you think about what was included? i.e. content
- Anything that you felt worked particularly well? Anything that could have been strengthened?
- Did it make sense to you as you did it?
- Was it easy to fit into your everyday life?

1. How did you use the ORBIT treatment?

- Did you use the parent sections?
- Did you view the child sections with your child?

1. How did you feel about your level of involvement in the treatment?

**Prompt**

- Did anything stop you getting involved?

1. How did it influence your approach to your child’s tics?

**Prompt**

- Any changes you made/strategies used?

Now some questions about how your child found using the ORBIT treatment:

*I think you said that you viewed the child sections with your child/your child completed ORBIT on their own?*

1. What do you feel about your child’s level of involvement with ORBIT treatment?

- Were there any barriers?

1. Overall, thinking about the child’s sections of ORBIT:

- Were there any sections that you think your child particularly enjoyed or engaged with and why?
- Were there any sections that did not engage your child or they found difficult? Why do you think this is?
- Do you think the therapy was too long/too short/just right?
- How did you feel about the level of the content for a child the age of yours?
- What about the rewards – how did you find them? Were they difficult to think of or stick too?
- Is there anything else you would like to add about the child’s section?

Thinking about the ORBIT therapy as a whole – parent and child sections:

1. Considering your expectations of the ORBIT treatment in terms of impact on your child:

- How effective did you expect it to be in reducing tics?
- Did you expect benefits in any other aspects of life?

1. In reality, what impact did the ORBIT treatment have:

- On your child’s tics?
- On any other areas of your child’s life?

1. What aspects of the treatment were particularly helpful?
2. Did you encounter any difficulties with the ORBIT treatment?

- If so, what were they?
- Were there technological difficulties? Did you manage to resolve these?
- Did that affect your overall view of the treatment?

1. Did you follow the ORBIT treatment exactly as it was structured?

- If not, how did you change it?

1. Would you change anything about the ORBIT treatment?

- What additional information, if any, should be included?
- Was anything included that was unnecessary?

1. How did you feel about communicating with (*name of therapist*)?

- How often did you contact them?
- Was it helpful?
- How did you contact them? Through email? Through ORBIT website? Phone?
- Which method did you prefer?

1. How did you feel about receiving treatment digitally?

- How would you have preferred?

Thank you for answering those questions. We only have a few questions left now and these will focus on the future of ORBIT:

**Future Direction**

1. If the ORBIT treatment is found to be effective are there any changes that we should make before it is routinely offered?
2. Are there other ways that you think could be used for delivering ORBIT?

- Skype? Webex?
- Other forms of technology?

1. Overall, how did you feel about the experience of participating in this trial?

- Would you recommend ORBIT treatment to other parents of children with tic disorders?

**Prompt** for clarification of response.

**End of questions**

That reaches the end of the interview and questions I wanted to ask you.

Thank you so much for giving me your time.

- Do you have anything else you wish to speak about that hasn’t been mentioned?
  - Let interviewee talk if they have anything else to add
  - If nothing else – then close interview

If you are okay to end the interview there, I will switch the Dictaphone off.

*Switch Dictaphone off*

**Debriefing**

- Ask how they are feeling – whether anything in the interview has troubled them or distressed them or if anything requires clarification
- They can email me if they have any follow up questions
- Thank them again, and ask if they are feeling okay to end interview here.

**THERAPIST INTERVIEW SCHEDULE**

**Preamble**

- Make sure refreshments available in room and that room is set-up ready for interview.
- Explain that:
- We are interested in individual experiences and thoughts about ORBIT, so please give honest responses, as both positive and negative feedback will help us improve the intervention. Explain that they will be asked questions relating to their involvement in ORBIT, their thoughts on the treatment, feedback they received, experience of supervision, and any recommendations they may have
- However, we combine all the data we collect to provide an overall picture of ORBIT and its implementation. Any comments in the report are attributed very generally, for example, “A therapist commented that…” All comments/opinions will be strictly confidential.

*Ask*: Do you have any initial questions?

**Ethics**

- Remind interviewee:
- The interview will take about 25 minutes
- You do not have to answer any questions that you are not comfortable with and there are no ‘right’ or ‘wrong’ answers
- You can stop at any time, no explanation needed
- If you need a comfort/loo break, please just say, that’s absolutely fine
- If any question doesn’t make sense, ask for an explanation

With your permission we are going to record the interview (audio only, on a Dictaphone) so that we can focus on what you are saying. This will be transcribed by a member of the research team or an approved company.

We remove any reference to any places, clinicians/therapists/family members that may give away yours (or others) identity during transcription.

The original transcription will be stored on an encrypted hard drive and no one other than immediate members of the research team can access this.

*Ask*: Do you have any questions about how we use your comments?

*Ask*: Is it okay to record the interview?

- If participant not satisfied: answer any questions they have. If they do not want to participate, thank them for their time and finish the interview at this point.

**Explain procedure**

I will begin the interview with my name, the date, and time - this is just to keep the recordings organised. All your details will be anonymised when the data is transcribed. The first part will be a little about yourself and your role in ORBIT, followed by general questions about the ORBIT project, then moving on to the ORBIT treatment more specifically and then ending with any recommendations and your overall experience of being involved in ORBIT.

*Ask*: Do you have any questions before we start?

*Ask*: Is it okay for me to start recording now?

*State researcher’s name, date, and time (for data management)*

I want to start by asking a bit about you:

**Background Questions**

1. Please briefly describe your professional background

- Profession
- If applicable, how long have you worked as a therapist with children/young people?

1. What was your particular role on the ORBIT trial?

- Were you involved in the creation of the ORBIT trial?
- Supervisor/supervisee?

1. What previous experience did you have that was relevant to your role as therapist/supervisor on ORBIT?

**Prompt**

- Training
- Education

1. What were your thoughts about online therapy before you began in this role?

Now I am going to ask you about your involvement in the trial:

**Questions about ORBIT as a trial**

1. How did you find out about the ORBIT trial?
2. Why did you get involved in this trial?

- How did you feel about being involved?

1. Are there any specific challenges for the therapist because the therapy is being delivered as part of a trial?

**Prompt**

- Keeping treatments separate/avoiding contamination
- Rigour of protocol
- Having sufficient time to adhere to the protocol
- Sense that you are offering a helpful, effective therapy in both arms of the trial

Thank you for these answers, that’s been really helpful. I’d now like to move on and ask you some questions on the role of the therapist and its subsequent demands:

**Therapist role and demands**

1. Can you tell me a bit about the role of the therapist in therapist assisted online treatment?

**Prompt**

- Any advantages/rewarding aspects to the role (e.g. convenience, job satisfaction etc.)?
- Any limitations (e.g. safety issues, feasibility etc.)?
- Any suggestions for overcoming identified limitations?

1. What personal skills/experience do you think are needed for a therapist to effectively implement the ORBIT intervention?

- How experienced do you think a therapist needs to be to deliver the intervention?
- Experience with digital interventions?
- Experience working with young people? Are there any specific challenges because the therapy is with young people?
- Prior clinical training?

1. Are there any training needs you can identify that may aid a therapist in delivering the intervention?

**Prompt**

- Tics and other neurodevelopmental conditions (OCD, ASD, ADHD)
- Training in assessments of tics
- Training in online therapy
- Training in ERP

1. How have you structured/managed this role alongside your other commitments?

- How many hours did you dedicate to ORBIT per day?
- How has the workload felt?
- Has anything felt particularly difficult/stressful?
- Were there any tools you used/found useful that helped manage your workload? E.g. excel sheets
- Are there any structural changes you can identify that would make the therapists’ role more effective/manageable?

1. Can you please share your experiences of the supervision you gave/received?

**Prompt**

- Quality of sessions – what went well/less well?
- Quantity of sessions
- Structure – how did the supervision work?
- Common issues that arose – general trial issues vs delivery of the intervention issues?

Now I would like to ask you some questions about delivering the specific treatments:

**Perceptions of delivering the different treatments**

1. Do you believe the online intervention is being delivered as planned (*describe what “as planned” means i.e. 10 chapters over 10 weeks, supporting patients by email etc*.)?

- If so, how? If not, why?
- Predictable outcomes?
- Any unanticipated consequences?

1. How do you see the role of the therapist in the ERP arm?

**Prompt**

- What sort of support do the children/parents need?
- Any examples where you felt the remote therapy went particularly well in the ERP arm?
- Any difficulties?

1. How do you see the role of the therapist in the Psychoeducation arm?

**Prompt**

- What sort of support do the children/parents need?
- Any examples where you felt the remote therapy went particularly well in the Psychoeducation arm?
- Any difficulties?

1. How do you feel about delivering online therapy?

- What are the benefits/limitations?

1. What feedback have you received from children/parents?

**Prompt**

- Benefits
- Problems encountered
- Frustrations
- Participant characteristics that influenced their feedback e.g. age/gender/comorbidities?

1. How often did you interact with participants online?

**Prompt**

- Daily/every other day/once a week/twice a week/more?
- What were the main types of comments you received?
- Was it manageable?
- What format were these interactions (F2F, phone, messages and comments on the worksheets via BiP)?
- Was the contact mainly with parents or children?
- Were there any difficulties in responding to participants?

1. How do you feel about the relationships you have developed with young people and their parents?

- Did the interactions feel meaningful?
- Was this more difficult to develop online than F2F (*if therapist has had prior experience of F2F therapy*)?
- Did this hinder/help the effectiveness of the therapy?

1. What is your view on the ERP arm and the things included in it?

**Prompt**

- Structure
- Content
- Use of videos/animation, quizzes
- Language/flow
- Too long/too short?

1. What is your view on the Psychoeducation arm and the things included in it?

**Prompt**

- Structure
- Content
- Detail
- Too little/too much information?

1. Would you change anything about the ERP/Psychoeducation programs?

- Additional information? Unnecessary information?

1. Why do you think children/parents may not wish to engage/persist with the interventions?

**Prompt**

- Barriers?
- Could we have done anything differently?
- How can we better engage them in future work?
- Characteristics of those who found it difficult to engage e.g. age/gender/comorbidities?

1. What do you believe were the main barriers to effectively implementing the interventions?

- Internal/external factors?
- Any solutions?

1. What do you think have been the overall effects of the intervention on participants?
2. Do you think face to face therapy is more effective than digital?

- If so, why?
- A combination of the two?
- Do you think face to face therapy may have given us a different outcome?

Thank you for answering those questions. We only have a few questions left now and these will focus on the future of ORBIT and your overall thoughts:

**Future Direction**

1. Is there anything else we could have done differently?
2. Overall, would you recommend the ORBIT intervention to children?

- Why?
- At what point of diagnosis/age?

**End of questions**

That reaches the end of the interview and questions I wanted to ask you.

Thank you very much for your time.

- Do you have anything else you wish to speak about that hasn’t been mentioned?
  - Let interviewee talk if they have anything else to add
  - If nothing else – then close interview

If you are okay to end the interview there, I will switch the Dictaphone off.

*Switch Dictaphone off*

**Debriefing**

- Ask how they are feeling and if anything requires clarification
- They can email me if they have any follow up questions/comments

Thank them again, and ask if they are feeling okay to end

**CLINICIAN INTERVIEW SCHEDULE**

**Preamble**

- Check that the interviewee has received the information sheet, understands the ORBIT project and his/her role in it
- Make sure refreshments available in room and that room is set-up ready for interview (*if interview is done face to face*).
- Explain that:
  - The aim of the ORBIT study was to investigate whether treatment delivered online can help children and young people with tics.
- The research team is speaking to a range of people involved in ORBIT e.g. children and parents who participated, therapists, and clinicians
- We are interested in individual experiences and thoughts about ORBIT, so please give honest responses, as both positive and negative feedback will help us improve the intervention. Explain that they will be asked questions relating to their involvement in the ORBIT trial, experiences with recruitment, and factors relating to their institution e.g. NHS
- However, we combine all the data we collect to provide an overall picture of ORBIT and its implementation and any comments in the report are attributed very generally, for example, “A clinician commented that…” All comments/opinions will be strictly confidential.

*Ask*: Do you have any initial questions about the project?

**Ethics**

- Remind interviewee:
- The interview will take about 20 minutes
- You do not have to answer any questions that you are not comfortable with and there are no ‘right’ or ‘wrong’ answers
- You can stop at any time, no explanation needed
- If you need a comfort/loo break, please just say, that’s absolutely fine
- If any question doesn’t make sense, ask for an explanation

With your permission we are going to record the interview (audio only, on a Dictaphone) so that we can focus on what you are saying. This will be transcribed by a member of the research team or an approved company.

We remove any reference to any places, therapists/family members that may give away yours (or others) identity during transcription.

The original transcription will be stored on an encrypted hard drive and no one other than immediate members of the research team can access this.

*Ask*: Do you have any questions about how we use your comments?

*Ask*: Is it okay to record the interview?

- If participant not satisfied: answer any questions they have. If they do not want to participate, thank them for their time and finish the interview at this point.

**Explain procedure**

I will begin the interview with my name, the date, and time - this is just to keep the recordings organised. All your details will be anonymised when the data is transcribed. The first part will be a little about yourself, followed by general questions about the ORBIT trial, moving on to your views on recruitment, and ending with institutional issues and future direction.

*Ask*: Do you have any questions before we start?

*Ask*: Is it okay for me to start recording now?

*State researcher’s name, date, and time (for data management)*

I want to start by asking some questions about you:

**Background Questions**

1. Please briefly describe your professional background

**Prompt** (if not covered)

- What is your job title?
- How long have you worked as a clinician with children/young people?
- How much contact do you normally have with children with tics?

1. What treatment recommendations would you normally prescribe for young people with tics?

**Prompt** (if not covered)

- Referral to a specialist therapist?
- Behavioural treatments and/or medication (referred for? Or given by themselves?)
- How confident are you in diagnosing tic disorders?

1. What % of children do you refer to behavioural treatments (BT)?

- What type of BT (CBIT/ERP/HRT)?

**Prompt**

- How easy is it to currently assess BT?
- What influences your decision to refer/give BT?
- How long is the waiting list?
- Do you believe BT is useful for tics?

Now I am going to ask you questions about being part of the ORBIT trial:

**Questions about ORBIT as a trial**

1. How did you find out about the ORBIT trial?
2. Why did you get involved in this trial?
3. How did you feel about being involved in this trial?
4. What were your expectations of the ORBIT trial?

- Did it sound like something that would be effective?
- Did you expect people to take part?

Thank you for these answers, that’s been really helpful. I’d now like to move on and ask about your thoughts on recruitment for the trial:

**Recruitment for ORBIT**

1. What was your experience of recruiting participants to the ORBIT trial?

**Prompt**

- Did it take a lot of time?
- How difficult was it to recruit to the trial?
- Were all the procedures (e.g. returning of consent to contact forms) clear?

1. Were there any factors that affected recruitment?
   **Prompt**

- Drivers to recruitment?
- Ability to offer a service for patients?
- Barriers?
- Employ any strategies to improve recruitment?

1. What factors influenced whether you approached a family about the trial?

**Prompt**

- Having the materials to hand?
- Remembering at the time?
- Characteristics of the family that you approached (e.g. engaged parents or those not currently in crises)?
- Availability of treatment options in your own clinic and locally

1. Why do you think children/parents may not have agreed to take part?
2. Why do you think children/parents may not have persisted with the intervention?

- How can we better engage children and families in future work?

1. Have you received any feedback from parents/children about the ORBIT trial?

- If so, what was it?

1. Do you think face to face therapy is more effective than digital?

- If so, why?

1. Overall, how do you feel about inviting patients to participate in studies external from your care?

**Prompt**

- Motivations for doing so?
- Is the fact the therapy is delivered externally a positive? Why?

Thank you for answering those questions. We only have a few questions left now and these will focus on institutional factors:

**Institutional factors**

1. What, if anything, have you/your clinic learnt from being involved in this trial?

- Anyone else from team involved?
- Outcomes?

1. How do you think the NHS could incorporate the ORBIT intervention into everyday practice?

**Prompt**

- Feasibility
- Benefits
- Obstacles

1. Do you think the NHS would be able to/willing to fund such a project?

- Costs versus benefits
- Good use of money?

**Future Direction**

1. Is there anything we could have done differently on this trial?
2. Overall, would you recommend the intervention to children?

- Why?
- At what point of diagnosis/age?

**End of questions**

That reaches the end of the interview and questions I wanted to ask you.

Thank you very much for your time.

- Do you have anything else you wish to speak about that hasn’t been mentioned?
  - Let interviewee talk if they have anything else to add
  - If nothing else – then close interview

If you are okay to end the interview there, I will switch the Dictaphone off.

*Switch Dictaphone off*

**Debriefing**

- They can email me if they have any follow up questions/comments
- Thank them again, and ask if they are feeling okay to end interview here.
