# Supplementary material for: Protocol for the Process Evaluation of the Online Remote Behavioural Intervention for Tics (ORBIT) randomized controlled trial for children and young people
Source: Trials. 2020 Jan 2;21:6. doi: 10.1186/s13063-019-3974-3 (PMC6941346; doi:10.1186/s13063-019-3974-3)
Supplement: Supplementary file 4 — Additional file 4. ORBIT Consent and Assent forms. [file 13063_2019_3974_MOESM4_ESM.docx]

**ASSENT FORM FOR YOUNG PEOPLE UNDER 16 YEARS**

**Centre Name:** Nottinghamshire Healthcare NHS Foundation Trust

**REC reference:** 18/NW/0079

**Participant Identification Number for this trial**:

**ASSENT FORM**

**Title of Project: Online Remote Behavioural Intervention for Tics (ORBIT)**

**Name of Researcher:**

**Please initial box**

1. I have read the information sheet dated 26 FEB 2018 (version 2.0) for the ORBIT study. I have discussed it with my mum/dad/carer and the researcher and I have asked questions.
2. I understand that I don’t have to take part and I can stop taking part any time. This is my choice and no-one will be upset with me if I stop.
3. I understand that the ORBIT team may look at my medical records and the data will be kept in a database both in England and in Sweden. This will be kept safe and only the research team will see my data.
4. I understand that the research team will write a report about the project. My name will not be mentioned in any reports.
5. I agree to my Doctor knowing that I am taking part in the ORBIT study.
6. The researcher might ask me to take part in an interview about my experiences of the ORBIT trial. I do not have to take part. If I agree to take part, the interview will be recorded but only the research team will know that I did the interview.
7. I agree to take part in the ORBIT study.

Name of young person Date Signature

Name of Person Date Signature

taking assent

When completed: 1 for participant; 1 for researcher site file; 1 (original) to be kept in medical notes.


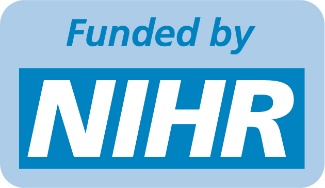


This research was funded by the NIHR Health Technology Assessment (ref 16/19/02). The views expressed are those of the author(s) and not necessarily those of the NHS, the NIHR or the Department of Health.

**CONSENT FORM FOR YOUNG PEOPLE 16 YEARS AND OVER**

**Centre Name:** Nottinghamshire Healthcare NHS Foundation Trust

**REC reference:** 18/NW/0079

**Participant Identification Number for this trial**:

**CONSENT FORM**

**Title of Project: Online Remote Behavioural Intervention for Tics (ORBIT)**

**Name of Researcher:**

**Please initial box**

1. I confirm that I have read the information sheet dated 25-MAY-2018 (version 3.0) for the
   above study. I have had the opportunity to consider the information, ask questions

and have had these answered satisfactorily.

1. I understand that my participation is voluntary and that I am free to withdraw at any time without giving any reason, without my medical care or legal rights being affected.
2. I understand that relevant sections of my medical notes and data collected during the study, may be looked at by individuals from the ORBIT team, from regulatory authorities or from the NHS Trust, where it is relevant to my taking part in this research. I give permission for these individuals to have access to my records. I understand this data will be stored in the UK (sealed envelopes) and Sweden (BiP and BASS) secure databases and servers.
3. I understand that the information collected about me will be used to support other research in the future, and may be shared anonymously with other researchers.
4. I agree to my General Practitioner being informed of my participation in the study.
5. I understand that I may be asked to take part in research interviews, which will be recorded and anonymous direct quotes from these interviews may be used in study reports.
6. I agree to take part in the above study.

Name of Participant Date Signature

Name of Person Date Signature

taking consent

When completed: 1 for participant; 1 for researcher site file; 1 (original) to be kept in medical notes.


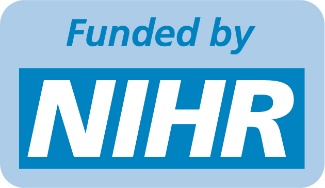


This research was funded by the NIHR Health Technology Assessment (ref 16/19/02). The views expressed are those of the author(s) and not necessarily those of the NHS, the NIHR or the Department of Health.

**CONSENT FORM FOR PARENTS/CARERS**

**Centre Name:** Nottinghamshire Healthcare NHS Foundation Trust

**REC reference:** 18/NW/0079

**Participant Identification Number for this trial**:

**CONSENT FORM**

**Title of Project: Online Remote Behavioural Intervention for Tics (ORBIT)**

**Name of Researcher:**

**Please initial box**

1. I confirm that I have read the information sheet dated 25-MAY-2018 (version 3.0) for the
   above study. I have had the opportunity to consider the information, ask questions

and have had these answered satisfactorily.

1. I understand that mine and my child’s participation is voluntary and that I am free to withdraw at any time without giving any reason, without my medical care or legal rights being affected.
2. I understand that relevant sections of my child’s medical notes and data collected during the study, may be looked at by individuals from the ORBIT team, from regulatory authorities or from the NHS Trust, where it is relevant to my taking part in this research. I give permission for these individuals to have access to my records. I understand this data will be stored in the UK (sealed envelopes) and Sweden (BiP and BASS) secure databases and servers.
3. I understand that the information collected about me and my child will be used to support other research in the future, and may be shared anonymously with other researchers.
4. I agree to my child’s General Practitioner being informed of our participation in the study.
5. I understand that I/my child may be asked to take part in research interviews, which will be recorded and anonymous direct quotes from these interviews may be used in study reports.
6. I agree for me and my child (named below) to take part in the above study.

Name of Parent/carer Date Signature

Name of child

Name of Person Date Signature

taking consent

When completed: 1 for participant; 1 for researcher site file; 1 (original) to be kept in medical notes.


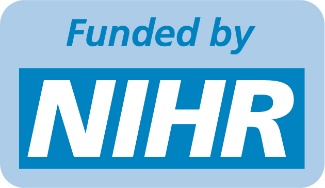


This research was funded by the NIHR Health Technology Assessment (ref 16/19/02). The views expressed are those of the author(s) and not necessarily those of the NHS, the NIHR or the Department of Health.
