# Supplementary material for: Protocol for the Process Evaluation of the Online Remote Behavioural Intervention for Tics (ORBIT) randomized controlled trial for children and young people
Source: Trials. 2020 Jan 2;21:6. doi: 10.1186/s13063-019-3974-3 (PMC6941346; doi:10.1186/s13063-019-3974-3)
Supplement: Supplementary file 5 — Additional file 5. Good Reporting of a Mixed Methods Study (GRAMMS) checklist. [file 13063_2019_3974_MOESM5_ESM.docx]

**Good Reporting of A Mixed Methods Study (GRAMMS)**

| **Guideline** | **Section: page** |
| --- | --- |
| Describe the justification for using a mixed methods approach to the research question | Design: p18  Strengths and limitations: p20 |
| Describe the design in terms of the purpose, priority and sequence of methods | Design: p18-19 |
| Describe each method in terms of sampling, data collection and analysis | Data collection: p11-14  Data analysis: p15-18 |
| Describe where integration has occurred, how it has occurred and who has participated in it | Design: p18-19 |
| Describe any limitation of one method associated with the present of the other method | Strengths and limitations: p20 |
| Describe any insights gained from mixing or integrating methods | Discussion: p19 |

*O'Cathain A, Murphy E, Nicholl J. The quality of mixed methods studies in health services research. J Health Serv Res Policy. 2008;13(2):92-98.*
